# Towards co-design of rehabilitation technologies: a collaborative approach to prioritize usability issues

**DOI:** 10.3389/fresc.2024.1302179

**Published:** 2024-02-21

**Authors:** K. Clanchy, J. Mitchell, K. Mulholland, E. Jurd, E. Kendall, D. G. Lloyd, D. Palipana, C. Pizzolato, C. Shirota

**Affiliations:** ^1^ School of Health Sciences and Social Work, Griffith University, Southport, QLD, Australia; ^2^ The Hopkins Centre, Menzies Health Institute Queensland, Griffith University, Nathan, QLD, Australia; ^3^ Griffith Centre of Biomedical and Rehabilitation Engineering, Menzies Health Institute Queensland, Griffith University, Southport, QLD, Australia; ^4^ Advanced Design and Prototyping Technologies Institute, Menzies Health Institute Queensland, Southport, QLD, Australia; ^5^ Emergency Department, Gold Coast University Hospital, Southport, QLD, Australia

**Keywords:** usability testing, technology, rehabilitation, disability, co-design

## Abstract

**Introduction:**

Early stakeholder engagement is critical to the successful development and translation of rehabilitation technologies, a pivotal step of which is usability testing with intended end-users. To this end, several methods employ end-user feedback to identify usability and implementation issues. However, the process of prioritizing identified issues seldom leverages the knowledge and expertise of the range of stakeholders who will ultimately affect the demand and supply of a device. This paper describes a novel method to prioritize end-user feedback using transdisciplinary stakeholder consultation and address it in subsequent product development. The proposed approach was demonstrated using a case study relating to the development of a novel technology for neural recovery after spinal cord injury.

**Method:**

Feedback from five individuals with chronic spinal cord injury was collected during two-hour usability evaluation sessions with a fully functional high-fidelity system prototype. A think-aloud and semi-structured interview protocol was used with each participant to identify usability and acceptability issues relating to the system in a 3-phase approach. Phase 1 involved extracting usability issues from think-aloud and semi-structured interview data. Phase 2 involved rating the usability issues based on their significance, technical feasibility, and implementation priority by relevant internal and external stakeholders. Finally, Phase 3 involved aggregating the usability issues according to design and implementation elements to facilitate solution generation, and these solutions were then raised as action tasks for future design iterations.

**Results:**

Sixty usability issues representing nine facets of usability were rated. Eighty percent of issues were rated to be of moderate to high significance, 83% were rated as being feasible to address, and 75% were rated as addressable using existing project resources. Fifty percent of the issues were rated to be a high priority for implementation. Evaluation of the grouped issues identified 21 tasks which were mapped to the product roadmap for integration into future design iterations.

**Discussion:**

This paper presents a method for meaningful transdisciplinary stakeholder engagement in rehabilitation technology development that can extended to other projects. Alongside a worked example, we offer practical considerations for others seeking to co-develop rehabilitation technologies.

## 1 Introduction

The role of technology in rehabilitation has attracted significant attention based on its potential to enhance therapeutic outcomes (1). For effective translation of rehabilitation technologies, the design and development process should be iterative and multidisciplinary. At a minimum, it should involve the stakeholders who will ultimately use or endorse the device (2–4). A critical step within this process involves end-users testing the usability of developed prototypes, where usability is defined as ease-of-use (5) or “*the extent to which a product can be used by specified users to achieve specified goals with effectiveness, efficiency and satisfaction in a specified context of use”* (6, page 68). Issues identified through usability testing with intended users, who likely think and act differently than technical experts, can inform design variations required to meet user needs and raise valuable considerations for the translation and implementation of the tested prototype in environments outside of the design and development space (7, 8). While several methods are available for identifying usability and implementation issues through the consultation of end-users (9–15), approaches to rating the criticality or priority of resolving these issues are traditionally undertaken less collaboratively.

The process of prioritizing identified issues is typically undertaken by technical experts based on objective user performance metrics (e.g., task success, time on task, errors, efficiency, and learnability) and frequency of issue occurrence (e.g., frequencies of issues within and across tasks, percentage of participants who experience a particular issue) (16). In parallel, the availability of technical expertise or project resources (e.g., personnel, funding, time) is considered (17). However, prioritization of issues in these ways lacks consideration of the quality of the user experience and does not account for the perspectives of pivotal stakeholders who have the potential to influence the demand and supply dynamics of technologies (16, 18). We instead argue that user feedback should be prioritized for integration into product design by additionally considering the impact of the design improvement on the cognitive and affective user experience (e.g., attitude towards device use, impact of device use on mood). User perceptions of the social and practical acceptability and utility of a device should also be considered during issue prioritization (5, 18, 19). Managing these numerous and sometimes competing priorities can be challenging, particularly when attempted in isolation by a single stakeholder group. Despite innovations including user-centred design, technical experts (e.g., engineers, designers) rarely have the lived experience necessary to represent users' point of view, while users rarely have the information or expertise necessary to understand the contextual factors impacting issue resolution (18, 19).

One way to address this challenge is to engage a wider range of technical and non-technical stakeholders in the prioritization of issues identified through usability testing (20, 21). Ongoing stakeholder consultation is consistent with co-design methodologies, in which diverse stakeholders are collaboratively engaged in design and development (2–4, 22). Through collaboration, stakeholders' experience and expertise can be leveraged to reconcile the numerous and competing priorities for implementation in future design variations. Collaborating in this way requires open communication and transparency in decision-making between stakeholder groups to ensure design iterations are clearly linked to user feedback. In this paper, we propose a novel method to prioritize user feedback through stakeholder consultation and to integrate this feedback into future product iterations in the context of rehabilitation technologies. In this approach, transdisciplinary stakeholder consultation refers to the inclusion of stakeholders in participatory problem-solving approaches that are applied to tangible, real-world problems (23). The proposed method is described and demonstrated using a case study based on the development of a technology for neural recovery after spinal cord injury. Significance, technical feasibility, and implementation priority ratings were collaboratively assigned to user-identified issues determined through a think-aloud and semi-structured interview protocol. Issues were subsequently grouped for the purpose of solution ideation and ratings were used to integrate solutions in the project's product roadmap. A worked example is included as part of the case study that demonstrates the process of identifying, prioritizing, and addressing one identified usability issue in the context of the product roadmap for the described technology.

## 2 Materials and methods

**Figure 1:**
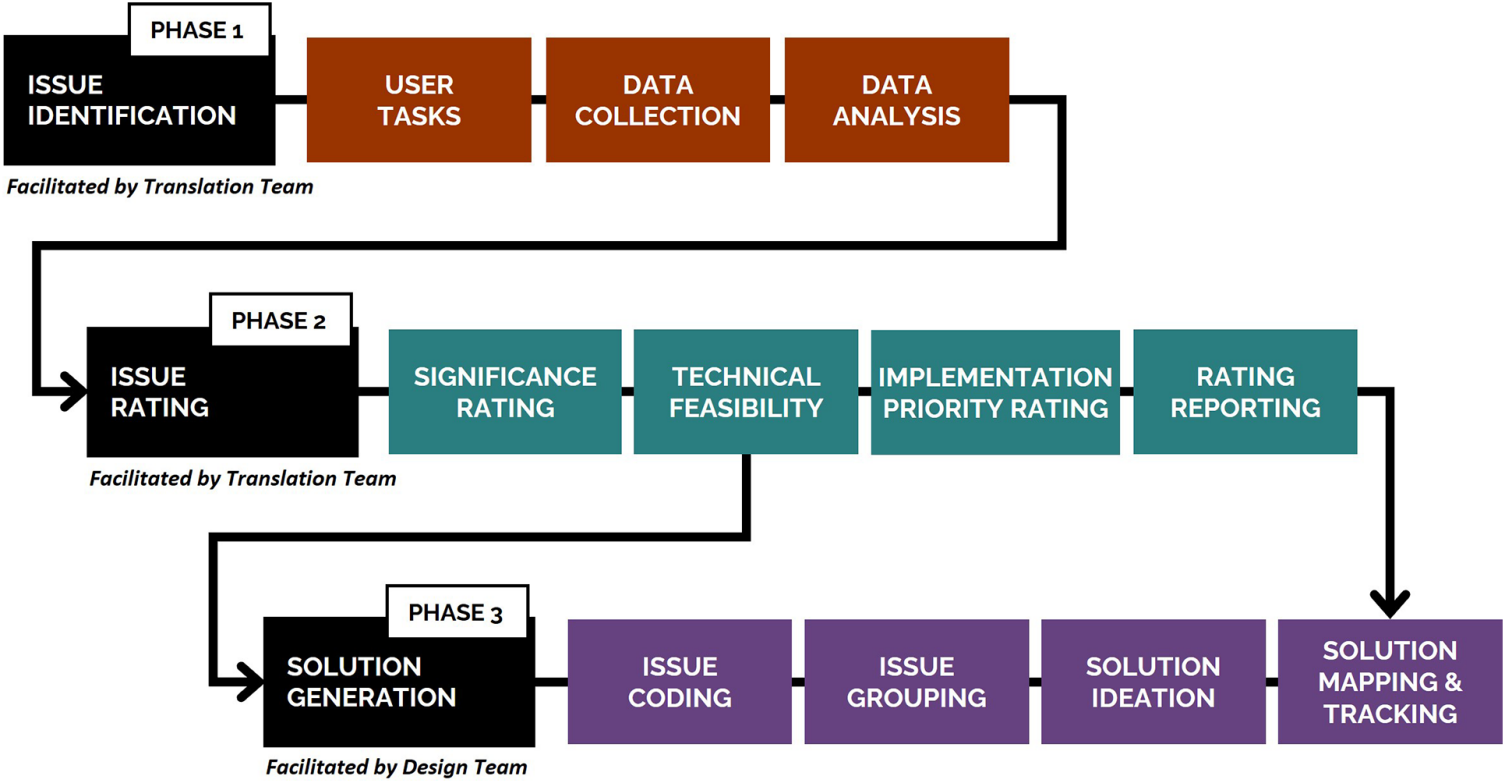
Three-phase process undertaken to collect and analyze usability data (Phase 1), rate identified usability issues (Phase 2), and generate solutions to improve system usability and acceptability (Phase 3). A detailed description of Phase 1 of the study, including the methods for the think-aloud and semi-structured interview protocols, has been published elsewhere (25).

The usability evaluation described in this paper forms one component of a larger research project developing a novel system for neural recovery after spinal cord injury (24). The project team comprised of three key bodies: internal Design and Translation Teams, and a Steering Committee of external stakeholders. A three-phase process (Figure 1) was undertaken collaboratively by these teams to collect and analyze usability data for issue identification (Phase 1), rate the significance, technical feasibility, and implementation priority of identified usability issues (Phase 2), and generate solutions to improve system usability and acceptability (Phase 3). Phases 2 and 3 are the focus of this paper. This process was developed collaboratively by-, and utilized the transdisciplinary expertise of- the Design and Translation Teams and Steering Committee members, which spanned lived experience of disability (including people with disability and formal and informal carers), health (including medical, allied health), neuroscience (including brain-computer interfaces and biomechanics), engineering (including robotics), design (including game and industrial), and policy (including legal policy and insurance).

This study was approved by the local ethics committee (Griffith University, reference number 2019/994).

### 2.1 Neurorehabilitation system

**Figure 2:**
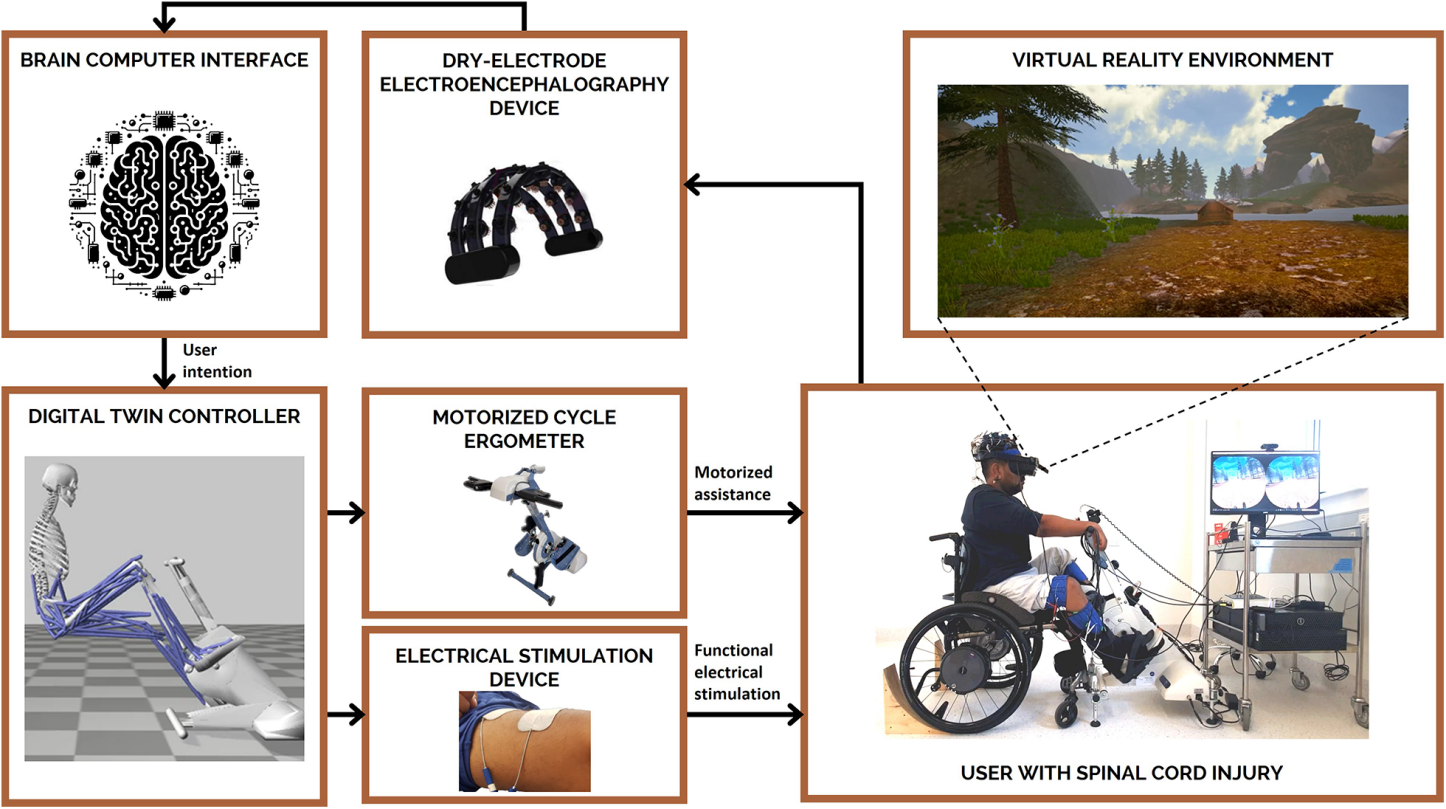
The system used in this case study combines non-invasive technologies to enable individuals with spinal cord injury to use their own thoughts (via a brain-computer interface; BCI) to control their own muscle(s) (via functional electrical stimulation; FES) and receive appropriate visual feedback (via virtual reality; VR) to engage in lower-limb rehabilitation (motorized cycle ergometer). Tasks included in usability testing were representative of a typical training session using the system prototype under the supervision of a trained clinician for a period of approximately two hours.

The system used as an example in the case study presented was a prototype of medical device with Technology Readiness Level 5, wherein the main technological components were integrated in a configuration similar to the final target application and tested in ecologically valid settings. The system followed a proposed framework for the establishment of a digital-twin based approach for interfacing rehabilitation devices to the individual's sensorimotor system (26, 27) and associated standards for the integration of this technology to Health Care (28). A representation of the system is included in Figure 2. In brief, the system enables a person with spinal cord injury to interface with rehabilitation technologies via a non-invasive brain-computer interface. The motor intention of the user is therefore converted, via a personalized digital twin of the person and the connected devices (26, 29), into control signals for the activation of muscle electrical stimulation and motorized assistance. Synchronous first-person view of the person, deployed into an engaging virtual environment, was also provided via immersive virtual reality. The rehabilitation system was developed following quality management system standards (ISO 13485) for medical devices. A key requirement of this standard is the documentation of design modifications, mandating a description of the change, its rationale, and its potential impact on the device. Stakeholder-identified issues during co-design offered valid justifications for these changes, which were meticulously recorded for traceability.

### 2.2 Phase 1: usability testing

**Figure 3:**
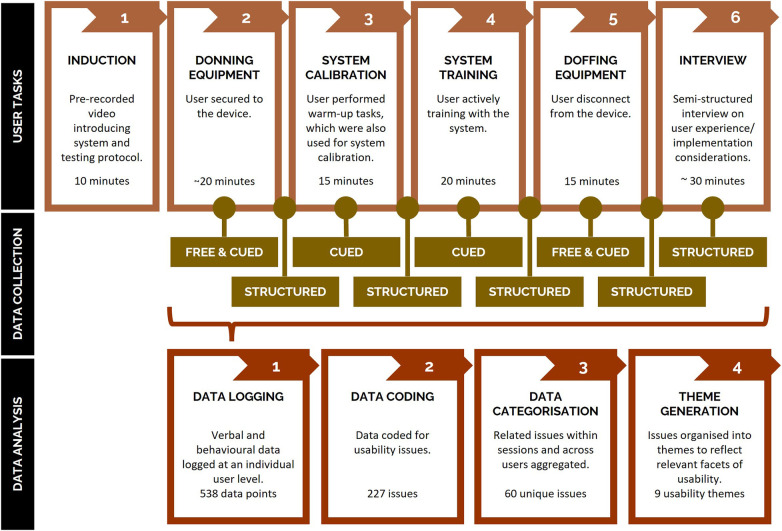
Overview of the process of identifying usability issues in Phase 1, including the tasks completed by users during testing sessions (top row; representative of a typical training session), think-aloud and semi-structured interview data collection methods utilized across tasks (middle row), and data analysis methods to extract usability issues from collected data (bottom row). Free = Free or unprompted thinking-aloud during system use. Cued = Cued or prompted thinking aloud during system use (e.g., 'can you tell me what you're thinking'). Structured = Structured or semi-structured questions asked to users after each phase. Device = neurorehabilitation system described in Section 2.1.

Individuals with a spinal cord injury who had experience using functional electrical stimulation and cycle ergometers were recruited using purposive sampling through the researchers' networks. Five individuals with a spinal cord injury (100% male; mean age = 32.6 years; mean time since injury = 7.3 years) were recruited to attend a typical training session using the system prototype under the supervision of a trained clinician in a university research lab located in Queensland, Australia for a period of approximately two hours. Participants were compensated for their time with an AUD $80 gift card and reimbursed for travel expenses. Concurrent and retrospective think-aloud methods, in combination with a semi-structured interview, were used to capture participants' thought processes and perceptions of the system (9, 11). In Phase 1, a four-step approach was utilized to extract usability issues from video-recorded think-aloud and semi-structured interview data: (1) data logging where data from the think-aloud and semi-structured interview protocol were logged at an individual participant level; (2) initial classification and coding where data were classified as an issue, positive, strategy or “other”, with similar data logged within and across participants; (3) higher-level categorization where codes were aggregated into higher-level categories based on their inter-relationships; and (4) theme generation where higher-level categories were organized into themes that reflected the facets of usability or the system component they related to. The application of this four-step approach resulted in the identification of 60 usability issues related to the design and implementation of the technology and representing nine themes or facets of usability (Table 1). Phase 1 was carried out by three members of the Translation Team, with support from technical staff. A detailed description of Phase 1 of the study (Figure 3), including the methods for the think-aloud and semi-structured interview protocols has been published elsewhere (25).

**Table 1:** Organization of the 60 identified usability issues related to the design and implementation of the technology and representing nine themes or facets of usability.

Facets of usability (themes)	Number of usability issues	Issue ID
Difficulties engaging with system training	13	#33–45
Comfort and positioning	12	#21–32
Safety and risks	9	#12–20
Knowledge and understanding	7	#1–7
User requirements (expertise and physical function)	6	#55–60
Commitment required to participate in a trial	5	#46–50
System issues and interruptions	4	#8–11
Outcome measurement approaches	3	#52–54
Accessibility of the physical space	1	#51

### 2.3 Phase 2: issue rating

**Figure 4:**
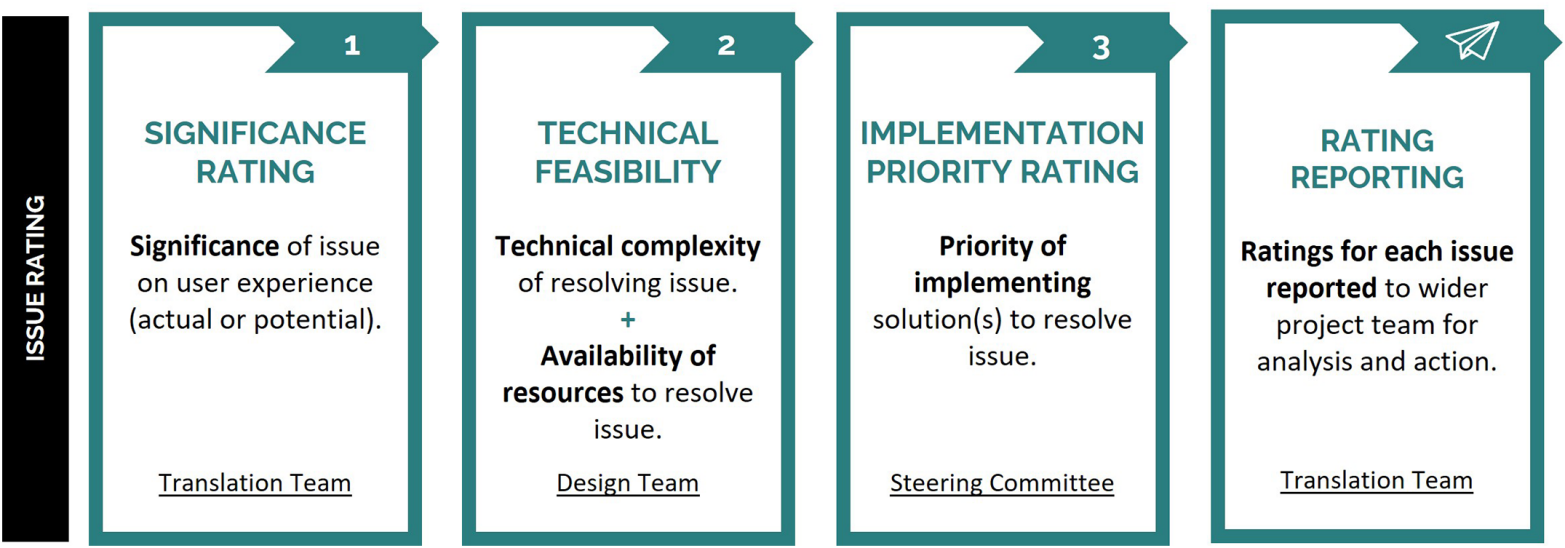
Overview of the process undertaken to prioritize user feedback in Phase 2. Each identified usability issue was provided with significance, technical feasibility, and implementation priority ratings, with final ratings reported to the Design Team for analysis and action.

**Figure 5:**
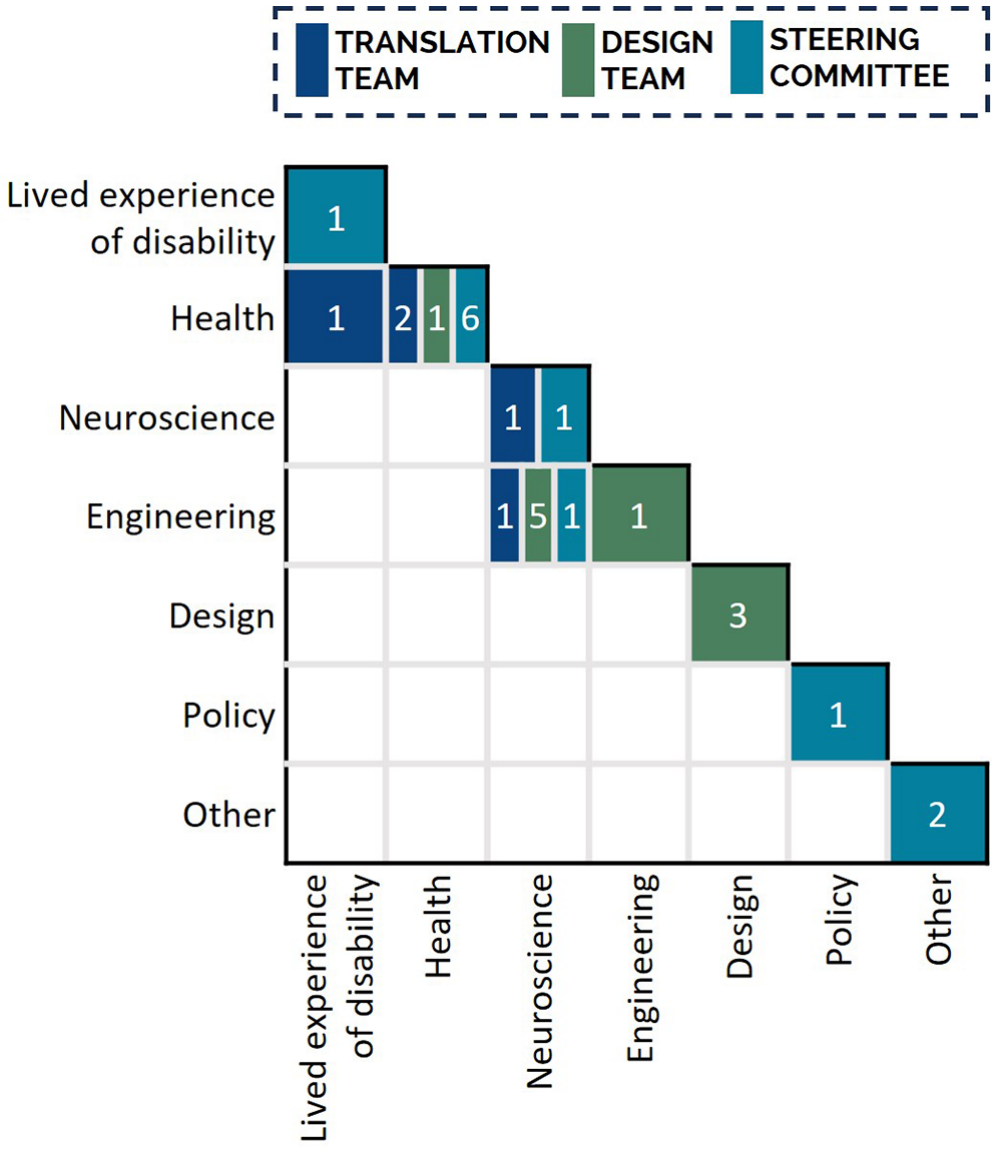
Expertise and lived experience of the Translation Team (*n* = 5), Design Team (*n* = 10), and Steering Committee (*n* = 12) members involved in usability issue rating. Nineteen team members indicated expertise in a single discipline; eight team members indicated expertise in two disciplines. All Translation Team members had expertise in disability and rehabilitation research. To interpret the Figure the reader is required to read both the column and row description to identify the expertise of the group of individuals included in the overall cell, then apply the color coding to determine the distribution of this expertise across the three stakeholder groups. For example, there were 7 individuals in total who identified joint expertise in neuroscience and engineering: 1 from the Translation Team, 5 from the Design Team, and 1 from the Steering Committee.

To prioritize usability issues for subsequent integration into the device's roadmap, issues passed through a three-step rating process in Phase 2 (Figure 4), which utilized the expertise and lived experience of members of the project team and external stakeholders. For the current study this included the project's Translation Team, Design Team, and Steering Committee (Figure 5). The rating process was led by three members of the Translation Team, two of whom facilitated the group-based significance and technical feasibility rating sessions. At the conclusion of this process, a report was provided to the Design Team detailing the identified usability issues and their respective ratings, with both visually mapped to facilitate interpretation and subsequent decision-making. Collated information relating to the rating scales is presented in Appendix 1.

#### 2.3.1 Significance rating

The significance of each usability issue encountered was rated by stakeholders with appropriate expertise to understand the significance of the issues on the experience of users with spinal cord injury and clinicians facilitating use. In the current study, this included five members of the project's Translation Team (Figure 4, Significance Rating), including the two members that facilitated the rating session. Translation Team members had lived experience of disability, as well as expertise in disability and rehabilitation research and related fields including health, neuroscience, and engineering (Figure 5). The significance rating was conducted during two online synchronous sessions. To inform their rating, the Translation Team were presented with all identified usability issues and the number of participants who encountered each issue.

The actual or potential significance of each usability issue on users' experience was rated on a four-point scale: minor (minor issue experienced by participant when using the system); moderate (moderate delay, frustration, or discomfort experienced by participant when using the system); severe (significant delay, frustration, or discomfort experienced by participant when using the system); and critical (participant was unable to use the system). In the current study, significance ratings were decided on using a consensus approach via discussion with all attending Translation Team members.

#### 2.3.2 Technical feasibility ratings

The technical feasibility of resolving each usability issue was determined through consultation with technical stakeholders who had the expertise to understand the technical complexity of addressing each issue and knowledge of the project resources available to address each issue. In the current study, this included ten members of the project's Design Team (Figure 4, Technical Complexity), who had diverse expertise across medical, neuroscience, engineering, and design fields (Figure 5). The Design Team were presented with the 60 usability issues and associated significance ratings during a single in-person session. Technical feasibility was comprised of two ratings: technical complexity and resource availability.

The technical complexity of resolving identified usability issues was rated on a four-point scale: minimal (resolving the issue is easy from a technical perspective); moderate (resolving the issue is moderately complex from a technical perspective); difficult (resolving the issue is difficult from a technical perspective); or not feasible (resolving the issue is not technically feasible with technology at that time). A binary resource availability rating (yes, no) was used to indicate whether the usability issue could be resolved with existing project resources (time, equipment, expertise, finances, etc.). In the current study, technical complexity and resource ratings were decided using a consensus approach via discussion with all attending Design Team members, which was facilitated by two members of the Translation Team.

#### 2.3.3 Implementation priority rating

The implementation priority of each issue was determined by stakeholders from a wide range of stakeholder groups relevant to the development and implementation of rehabilitation systems. Stakeholders were external to the system development to allow for more independent evaluation of priority. In the current study, twelve external stakeholders comprising the project's Steering Committee were consulted to provide an independent evaluation of usability issues by assigning an implementation priority rating (Figure 4, Implementation Priority Rating). Steering Committee members represented a diverse set of stakeholders in rehabilitation technology, with lived experience of disability and expertise in the fields of health, neuroscience, engineering, and policy (Figure 5). Usability issues were summarized alongside their significance and technical feasibility ratings for presentation to the Steering Committee. Due to their availability, each Steering Committee member provided an independent implementation priority rating via an online survey (programmed using the platform REDCap, RRID:SCR_003445).

The priority of implementing solutions to resolve identified usability issues was rated on a four-point scale: not a priority (resolving issue is unnecessary and/or unfeasible); low priority (issue to be resolved over the long-term i.e., after the next 9 months and using future project funding); mid priority (issue to be resolved in the short- to mid-term i.e., in the next 6–9 months and before completion of current project funding); or high priority (issue to be resolved immediately or in the short-term i.e., in the next 6 months). In the current study, final implementation priority ratings were determined via the median rating for each usability issue.

### 2.4 Phase 3: solution generation

**Figure 6:**
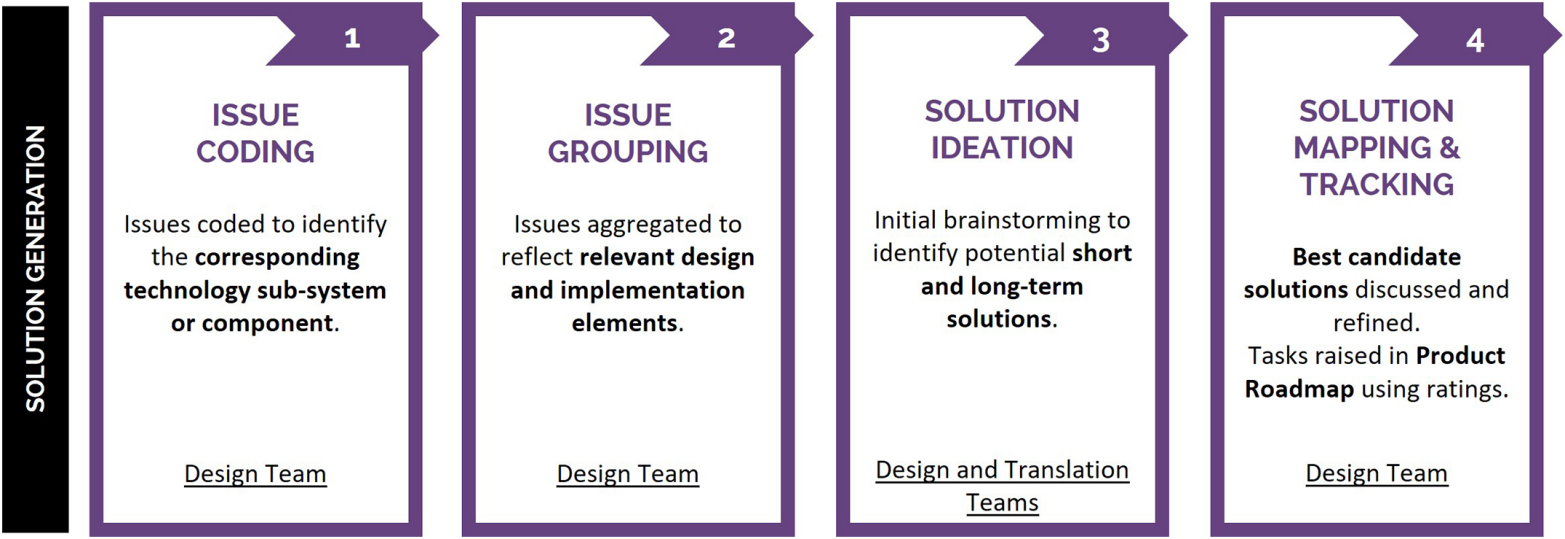
Phase 3 solution generation workflow, employed to assess the 60 identified usability issues. Issues were coded and grouped to reflect affected system design and implementation elements and subsequently facilitate solution generation, with resulting tasks raised in the product roadmap for future design iterations.

Following the technical feasibility rating process in Phase 2, a process was undertaken to determine potential solutions to the identified issues and prioritize these solutions for implementation in the product roadmap. In the current study, the Design Team began a four-step workflow (Figure 6) to review each usability issue and subsequently raise action tasks in the product roadmap for future integration into the system. Phase 3 was led by two members of the Design Team, one of which participated in Phase 2 ratings.

The Design Team analyzed the 60 usability issues, applying a codification system to identify the technology's corresponding sub-system or component (Figure 6, Issue Coding). Codification facilitated subsequent consolidation of usability issues into design and implementation elements associated with user-feedback (Figure 6, Issue Grouping), which were the focus of solution ideation. Over the course of five weeks, the Design Team held three, three-hour breakout workshops wherein potential short- and long-term solutions were ideated through open discussion with members of the Design and Translation Teams (Figure 6, Solution Ideation). Workshop attendees included one individual with lived experience of disability and members with diverse expertise across the fields of health, neuroscience, engineering, and design. Initial brainstorming identified potential short- and long-term solutions, with members from both the Design (*n* = 12, two of which had not participated in Phase 2 ratings) and Translation (*n* = 2, who had facilitated Phase 2 ratings) Teams contributing. All ideas were recorded. Design Team subject matter experts then refined best candidate solutions for their delegated design and implementation elements. Best candidate solutions were mapped into the product roadmap for integration into future iterations of the technology (Figure 6, Solution Mapping and Tracking). Product roadmap tasks were finalized after Phase 2 ratings were reported and therefore were informed by the significance, technical feasibility, and implementation priority ratings. Tasks associated with high significance ratings were prioritized for initial development, specifically usability issues deemed to impact user safety, comfort, and experience. Within this subgroup of usability issues, relevant technical complexity, and resource availability factors—such as the number of staff working on a sub-system, their existing workload, time availability, the number and complexity of existing tasks slated for priority development at the time, etc.—were taken into consideration when defining the implementation timeline for product roadmap tasks. Implementation ratings provided independent, external stakeholder input into prioritizing resolution of usability issues, used to further inform/support product roadmap timeline.

## 3 Results

### 3.1 Issue ranking and ranking interpretation

**Figure 7:**
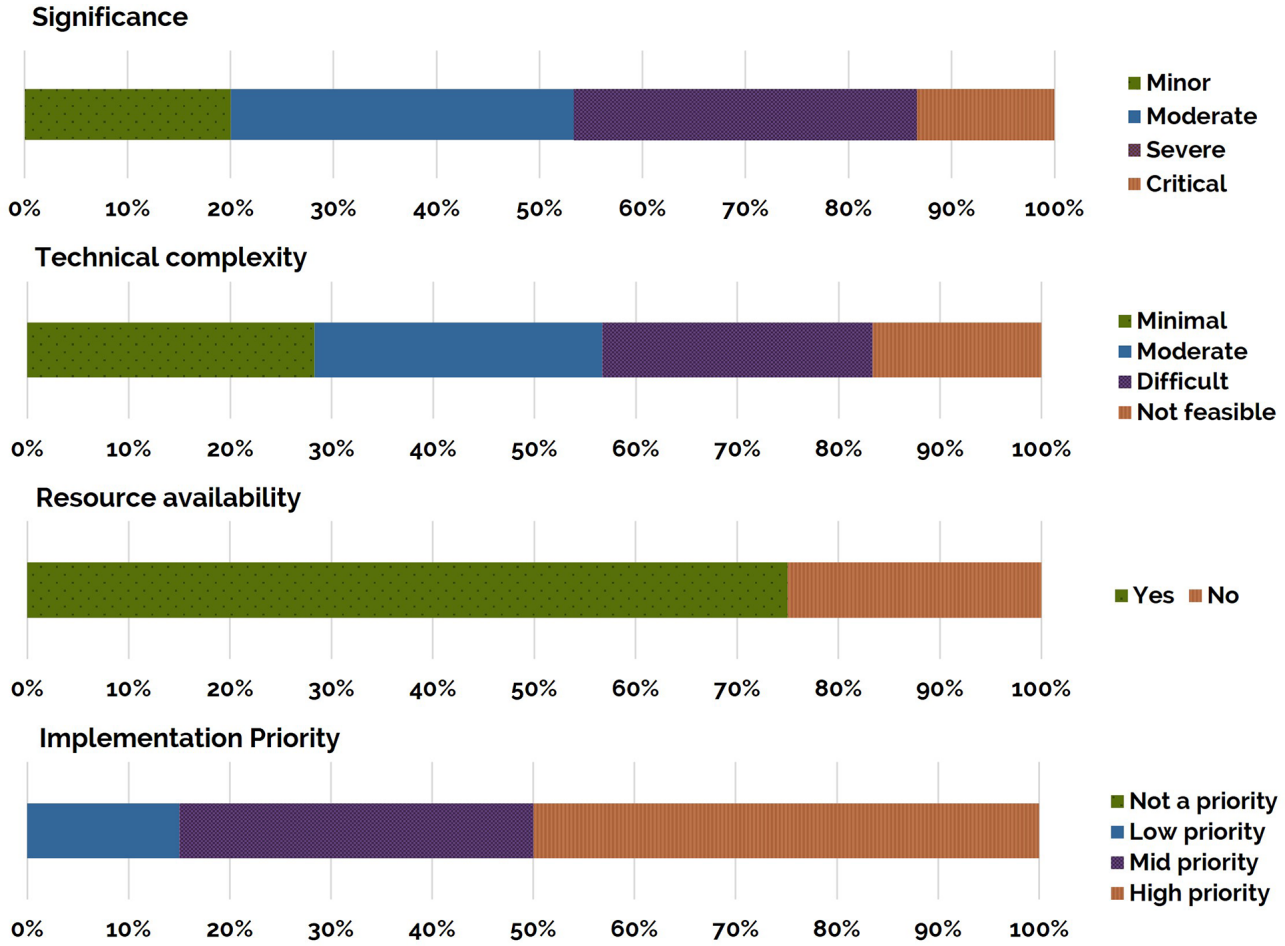
Ratings for the identified 60 usability issues: significance, technical complexity, resource availability and implementation priority (top to bottom). Each bar presents the percentage of the total number of issues identified at each rating. Rating descriptions are presented in Appendix 1.

Eighty percent of the 60 identified usability issues in the current study were rated as moderate significance or higher, with the most frequently occurring ratings being severe and moderate (Figure 7). Technical complexity ratings were distributed relatively equally across the ratings of minimal, moderate, and difficult. Seventeen percent of usability issues were rated as not being feasible to address. Seventy-five percent of the identified usability issues were rated as being able to be addressed with the existing project resources. Fifty percent of the identified usability issues were rated as being of high priority, requiring immediate resolution within the following 6-months of the project. No usability issues were classified as “not a priority.”

**Figure 8:**
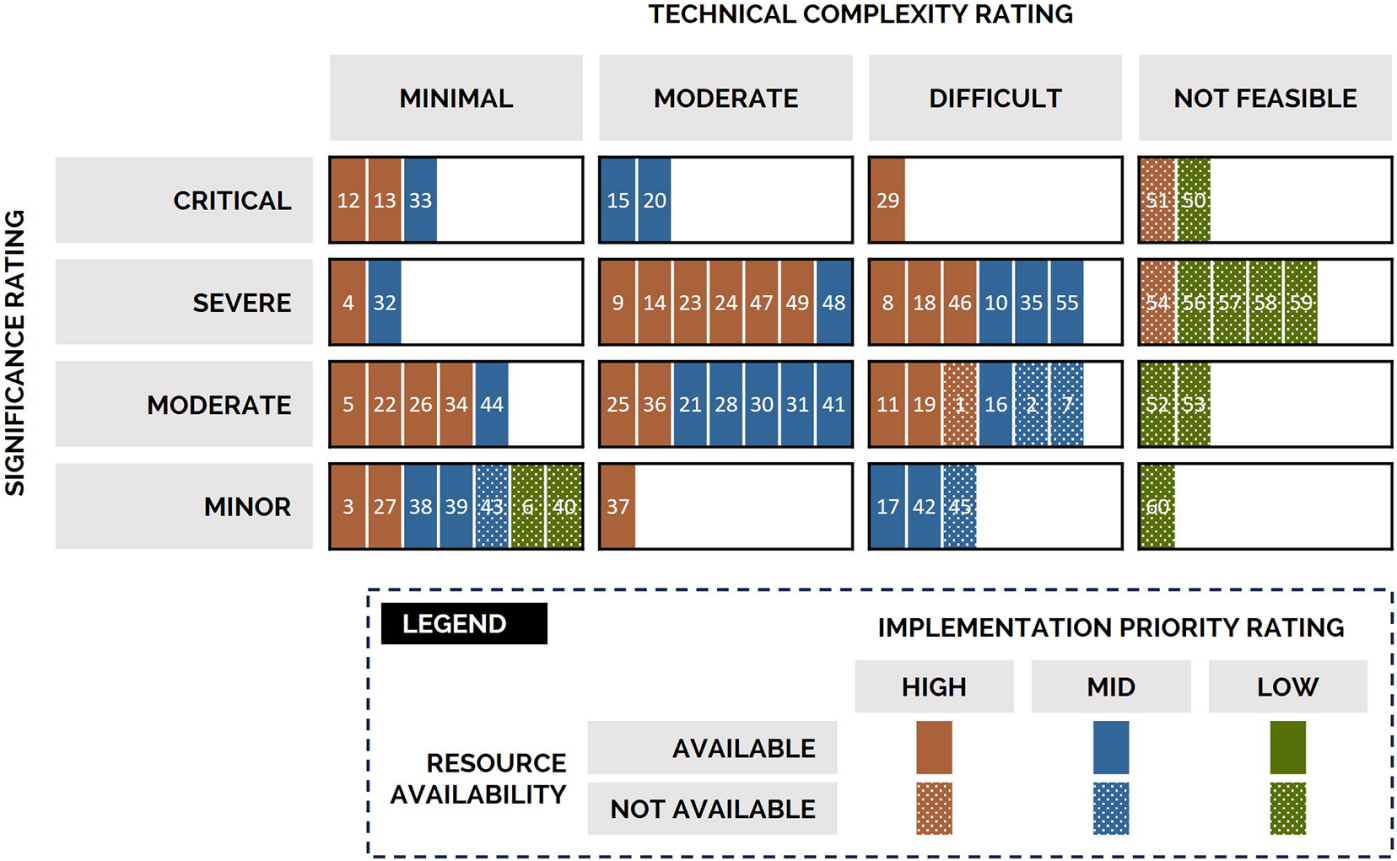
Mapping of usability issues according to their significance (rows), technical complexity (columns), resources (patterning), and implementation priority (color) ratings. Issue numbers are provided solely as indicators as specific issues are not of relevance here. Usability issues would be prioritized from the top-left box (critical significance, minimal technical complexity), with issues represented in solid brown fill (high implementation priority, resources available) prioritized first. No usability issues were classified as “not a priority.”.

Usability issues were organized and visually mapped on the basis of their significance, technical complexity, resource availability, and implementation priority ratings (Figure 8). Usability issues increase in significance from the bottom of the map to the top and increase in technical complexity from left to right. Usability issues would be prioritized from the top-left box, with issues represented in solid brown fill prioritized first. Of the 60 usability issues, 33% were rated as high priority, of notable significance to the user experience (moderate to critical), and technically feasible to resolve (minimal to difficult complexity with available resources). For example—usability issue 13 was identified as having a critical significance (top row) and minimal technical complexity (left-most column), with high implementation priority (brown fill) and available resources to address (solid fill)—therefore addressing this issue should be prioritized.

### 3.2 Integrating usability issues into the product roadmap

**Figure 9:**
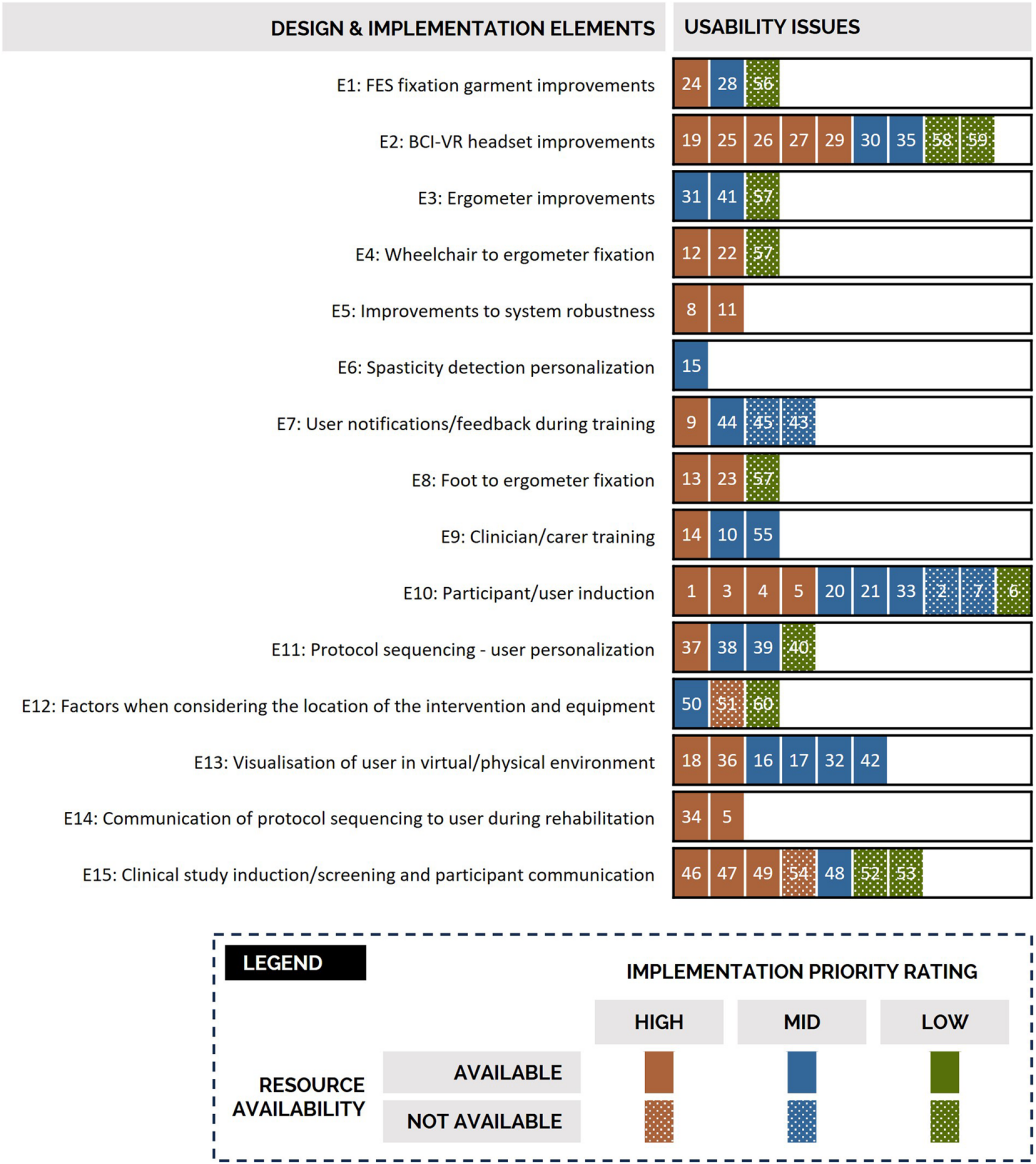
Grouping of the 60 usability issues into 15 design and implementation elements to support solution generation. Each issue has its associated implementation priority and resource availability indicated using the formatting indicated in the provided legend. For example, usability issues 24, 28 and 56 were identified as relating to the design and implementation element relating to functional electrical fixation garment improvements. Issue 24 was identified as high implementation priority, 28 as mid, and 56 as low. There were resources available within the project to address issues 24 and 28. FES = functional electrical stimulation; BCI = brain computer interface; VR = virtual reality.

The 60 usability issues were coded to the technology's corresponding sub-system or component and then aggregated into 15 design and implementation elements (Figure 9) to facilitate solution generation. Several issues related to more than one design or implementation element (e.g., issue 57 related to E3, E4, and E8). After completion of the breakout workshops and refinement of best candidate solutions, 21 tasks were raised within the technology's product. Of the 21 tasks, six were deemed to be technically feasible to address with currently available project resources (i.e., staff with appropriate expertise had access to required technologies and capacity to implement planned solutions). These six tasks were therefore actioned and integrated into the succeeding two technology iterations, the development and release of which ran on 3-monthly cycles. In parallel, development activities were initiated for an additional nine product roadmap tasks. These nine tasks had higher technical complexity and/or resources were only expected to become available to fully implement solutions in future development releases (i.e., staff with appropriate expertise were already engaged in previously planned development activities and/or implementing solutions to other roadmap tasks). The remaining six product roadmap tasks await resource availability, completion of predecessor tasks, and/or maturation of associated sub-systems prior to action and are therefore planned to be implemented using future project funding. For illustrative purposes, a worked example following the identification, prioritization, and resolution of issue 9 through two of the 21 tasks is provided below.

### 3.3 Worked example of issue 9

Usability issue 9 related to the potential for system interruptions to confuse users and was encountered by two out of the five individuals with a spinal cord injury during usability testing. This issue was rated to be of severe significance, highly impacting user comprehension and experience as participants identified misattributing system interruptions to their own actions (Figure 4, Significance Rating). While the operating clinician receives system state notifications via a graphical user interface, this information was not displayed to the user within the virtual reality environment. Absence of clear and timely information provided to the user about the cause of system interruptions was identified by the Design Team as contributing to user confusion. The issue was rated of moderate technical complexity to address, due to multiple system components that could cause changes in the functioning of the system (e.g., excessive negative torque being produced by the user during cycling beyond safety limits due to spasticity, or a electrode no longer in contact with the user's skin) (Figure 4, Technical Feasibility). There were deemed sufficient resources within the existing project to address the issue (i.e., staff with appropriate expertise had capacity), due to the potential of addressing the issue through a short-term solution (i.e., presenting real-time information to the user about the system being interrupted). On the basis of its significance and technical feasibility ratings, issue 9 was rated by the Steering Committee as having high implementation priority, requiring resolution within the next 6-months (Figure 4, Implementation Priority Rating).

The Design Team coded usability issue 9 as a system/protocol operation issue (Figure 6, Issue Coding), then grouped it under the “user notification/feedback during training” (E7) design element (Figure 6, Issue Grouping). Following solution ideation (Figure 6, Solution Ideation), two tasks were raised within the product roadmap (Figure 6, Solution Mapping and Tracking). Due to the severe significance rating of the underlying usability issue and associated high implementation priority rating, tasks were raised for immediate action.

**Figure 10:**
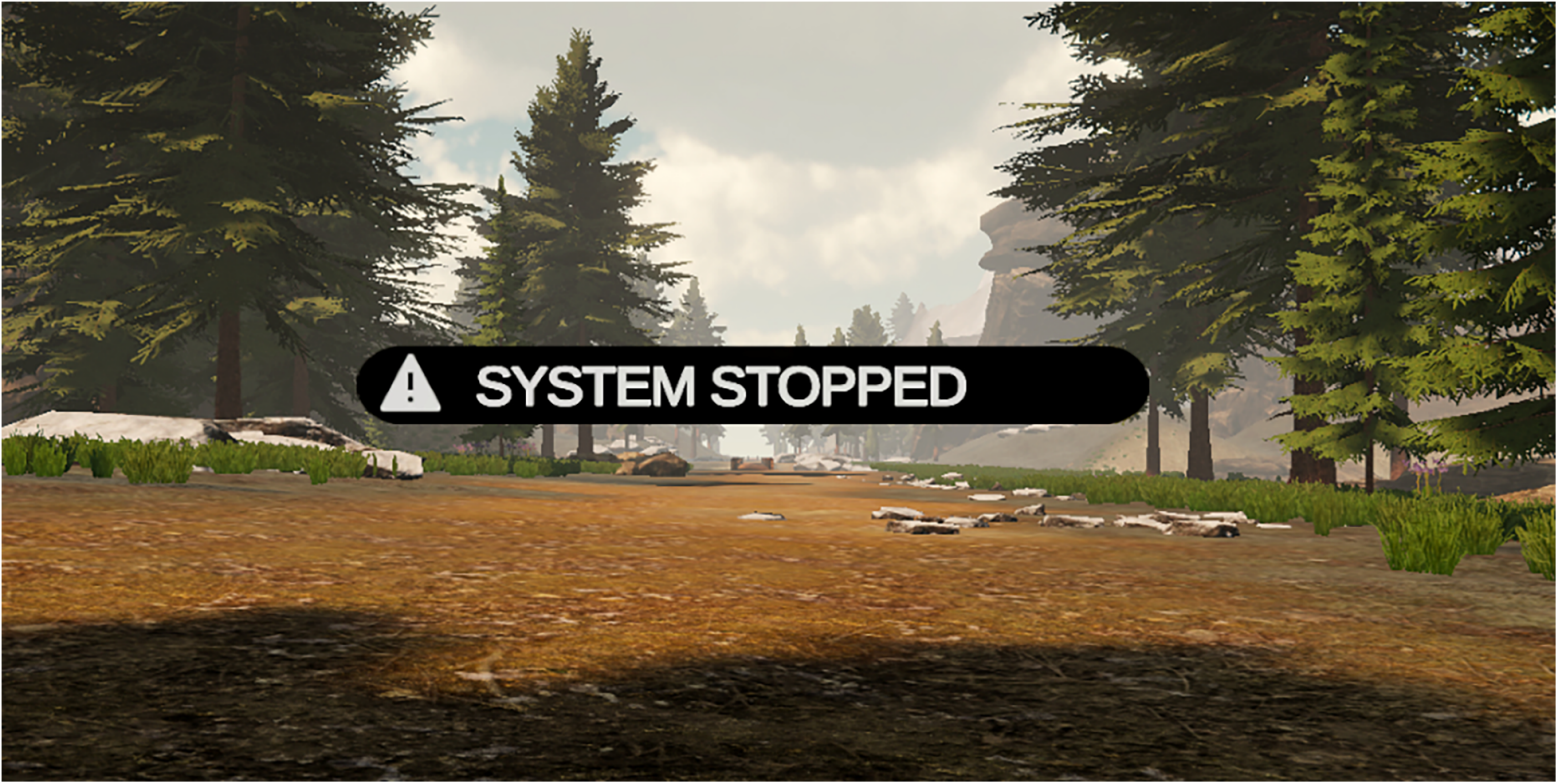
New system status notification implemented in response to usability testing. To reduce confusion to the users during potential system interruptions, the depicted notification will be provided to the user displayed via virtual reality during system interruptions.

The first task targeted improved visual system notifications for the user within the virtual reality environment. The notifications would be provided to the user during system interruption, e.g., in response to spasticity triggering the safety monitoring system (Figure 10). The first task scheduled for integration within the subsequent design iteration.

The second product roadmap task related to integrating sound notifications for the user. These sounds indicate not only the system status, but also session progress, providing additional context to users so they can better understand the source of interruptions and lessen confusion. The second task required modifications to interfacing sub-systems. Accordingly, the second task was scheduled for integration within a later design iteration.

## 4 Discussion

Usability testing can raise numerous and sometimes competing priorities that can be challenging for technical experts alone to manage. In this paper, we propose a collaborative method to prioritize user-identified issues, and subsequently integrate solutions to these issues into a device's product roadmap. We presented a case study of a novel system for neural recovery after spinal cord injury, where the project's Translation and Design Teams and Steering Committee iteratively worked on 60 issues previously identified by end-users.

Single-stakeholder approaches to evaluating usability may easily overlook the diverse perspectives of various relevant rehabilitation stakeholders, leading to an unbalanced assessment of a device. Collaboration between technical and non-technical stakeholders in the proposed usability evaluation method promotes consideration of objective and subjective user experiences, verification of pre-conceived expectations, reconciliation of competing design and implementation priorities, and transparency in decision-making. To promote collaboration with stakeholders, we applied a multi-phase method wherein three separate transdisciplinary groups applied their lived experience and expertise to collaboratively assign ratings to identified usability issues. The significance of usability issues was rated by translation experts, which was provided to design and development experts who rated technical feasibility, both of which were provided to external stakeholders to consider when rating the implementation priority of potential solutions. Significance and technical feasibility ratings were decided through consensus, which allowed for participating members' different experiences and expertise to be considered prior to a final rating being assigned. In contrast, due to practical issues related to the limited availability of the project's external stakeholders, each Steering Committee member provided an implementation priority rating independently and in consideration of their own experiences and expertise only. To reduce the subjectivity of the implementation priority ratings, tangible criteria relevant to the project for each rating were specified, e.g., a mid-priority implementation issue needs to be resolved within 6–9 months prior to the completion of the funded project, and the median rating across all Steering Committee members was assigned as the final implementation priority. This transdisciplinary approach engaged individuals in the fields of health, policy, engineering, design, and neuroscience, as well as individuals with a spinal cord injury. Although stakeholder engagement can be costly and time intensive, it was considered important to provide a broader perspective to the implementation priority of usability issues, that was less biased by existing priorities and demands within the project.

In our approach, the Translation Team considered the number of users who experienced each usability issue in conjunction with the proposed impact of the issue on a user (e.g., discomfort and/or inability to use the system) in assigning significance ratings. The implementation priority of resolving each issue was evaluated by the Steering Committee using this information in the context of the technical complexity of resolving the issues and resources available within the project. Issue ratings were visually represented to guide the implementation of tasks to resolve issues within the technology design process. Previously, data-driven approaches have been used to objectively quantify the significance or severity of issues. Abrantes and colleagues (18) propose a classification method to organize issues into four quadrants, where assigning a quadrant is based on an interaction between the criticality of the task being completed and relevancy of the problem. However, as the criticality rating is based on task completion, rather than the impact on the user, it can be argued that this method provides a limited understanding of user experience. Hassenzahl (30) explored a problem-handling time metric approach to severity estimates, in which the amount of time users spent dealing with interaction issues directly indicated the severity of the problem, regardless of frequency. In this data-driven approach the experiential elements of usability are ignored, e.g., an issue that creates a high level of discomfort over a smaller period may be prioritized lower than an issue with a moderate level of discomfort over a longer period. Sharon and colleagues (31) presented a 3-level scale of issue severity, influenced by the frequency of the issue e.g., if an issue occurs in more than 10 users it is automatically classified as a high-severity problem regardless of the significance of its impact on their individual experiences. However, this approach limits the understanding of the significance of issues experienced by single users (*n* = 1), which by this system would be considered as “irrelevant” or of low significance (14). Considering the small number of participants commonly recruited for usability testing in academic research, we propose that frequencies alone should not be used to prioritize or dismiss usability issues. Rather, we suggest infrequently occurring issues serve as a starting point for further investigation of the usability, utility, and acceptability of the device across a wider user group of representative users.

Usability issues integrated into the product roadmap in our case study were influenced by implementation priorities identified by the external stakeholders that were the Steering Committee, as well as factors specific to the Design Team's workflow. For example, opportunistic resource availability (e.g., small research projects, internships, funding opportunities) may influence the order in which identified usability issues are able to be addressed, e.g., funding becomes available for a small research project that could address an issue with a low implementation priority. Resource-related factors, including the availability of staff to work on multiple usability issues relating to a particular sub-system and/or the number and complexity of existing tasks identified for priority development, also influences the order in which priorities can be addressed. Additionally, some of the issues raised during the usability testing were identified during parallel pilot testing of the technical aspects of the prototype and addressed prior to the end of the rating process. In these instances, implementation priority ratings served to confirm assumptions held by project designers and provided valuable insight into the importance of these tasks. Mapping and tracking of solutions were led by the projects' Design Team. In co-design, all stakeholders engaged in a project should be actively involved in and share decision-making throughout development (2–4). However, in practicality, not all stakeholder groups are involved in all elements of day-to-day decision making. It is therefore essential that transparency in, and communication of, the decision-making process is maintained (e.g., through the mapping of tasks in a product roadmap) to ensure that the final solution reflects the opinions of multiple stakeholder groups.

### 4.1 Limitations and future directions

There are several practical considerations or limitations with our approach that warrant notice. Separate to usability session participants, two individuals with lived experience of spinal cord injury were involved in assigning the significance and implementation priority ratings (one individual per rating), and one individual with lived experience of spinal cord injury was involved in solution generation workshops. We note that inviting the original participants of the usability trial to participate in the significance and implementation priority rating sessions alongside project team members would have increased the voice of individuals with lived experience of spinal cord injury and, therefore, enhanced the robustness of the overall process. Consultation can be a timely process, dependent on the availability of stakeholders outside of the project or the organization, therefore in some instances, usability issues were identified in parallel by chance and addressed prior to ratings being made available. Approaches like instant data analysis, in which usability sessions are held on a single day and followed by a single brainstorming session in which as many issues remembered are recorded and mapped, may help to expedite the process (14, 15). Issues were categorized twice in our approach, once to indicate the associated facet of usability (in Phase 1), and once according to the design and implementation elements (in Phase 3). A more collaborative approach from the onset between the Design and Translation Teams in the organization and categorization of the data may have improved the efficiency of this analysis process. To further enhance the robustness of the methods detailed in this study, we encourage future work that uses collaborative approaches to streamline the organization and categorization of data (i.e., to ensure that initial data categorization is able to facilitate both issue rating and solution generation) and invites usability trial participants with lived experience of disability to participate in rating sessions alongside project team members (i.e., in a way that means power is shared during decision-making).

## 5 Conclusion

In this study, we introduced an approach for the prioritization of usability issues highlighted by end-users during the evaluation of a novel rehabilitation technology. The distinguishing strength of this approach is its embrace of transdisciplinary collaboration, amplified by the independent prioritization executed by an external stakeholder group, enriched by a spectrum of pertinent experiences and expertise in the field of technology for rehabilitation. This approach embeds co-production principles such as including all perspectives and skills, respecting and valuing the knowledge of all those working together on the research, and sharing power. We have provided a detailed example to elucidate how significance, technical feasibility, and implementation priority ratings can be practically utilized to transparently inform future design iterations. We also discuss recommendations for how the described usability issue rating method could be improved for efficiency and application in other testing environments.

## Data Availability

The datasets presented in this article are not readily available as the technology discussed is under active development and full data disclosure would violate intellectual property agreements. Requests for access to redacted transcripts of interviews should be directed to KC, k.clanchy@griffith.edu.au. Requests to access the datasets should be directed to KC, k.clanchy@griffith.edu.au.

## Appendix 1: Rating scales applied

**Table:**

Rating		Scale
Significance rating		Minor = minor issue experienced by participant when using the system Moderate = moderate delay, frustration, or discomfort experienced by participant when using the system Severe = significant delay, frustration, or discomfort experienced by participant when using the system Critical = participant was unable to use the system
Technical feasibility	Technical complexity	Minimal = resolving the issue is easy from a technical perspective Moderate = resolving the issue is moderately technically complex Difficult = resolving the issue is difficult from a technical perspective Not feasible = resolving the issue is not technically feasible
	Resource availability	Yes = the issue can be resolved with the existing project resources (time, equipment, expertise, financial costs) No = the issue cannot be resolved with the existing project resources (time, equipment, expertise, financial costs)
Implementation priority rating		Not a priority = resolving issue is unnecessary and/or unfeasible Low priority = issue to be resolved over the long-term i.e., after the next 9 months and using future project funding Mid priority = issue to be resolved in the short- to mid-term i.e., in the next 6–9 months and before completion of current project funding High priority = issue to be resolved immediately or in the short-term i.e., in the next 6 months
